# Two Cases of Pneumatosis Intestinalis during Cetuximab Therapy for Advanced Head and Neck Cancer

**DOI:** 10.1155/2015/214236

**Published:** 2015-07-29

**Authors:** James A. Miller, Daniel J. Ford, Mohamed S. Ahmed, Thom R. Loree

**Affiliations:** ^1^The Center for Oncology Care, Erie County Medical Center, 462 Grider Street, ACC Building 2nd Floor, Buffalo, NY 14215, USA; ^2^School of Medicine and Biomedical Sciences, University at Buffalo, Buffalo, NY 14214, USA; ^3^Department of Head & Neck and Plastic & Reconstructive Surgery, Erie County Medical Center, 462 Grider Street, ACC Building 2nd Floor, Buffalo, NY 14215, USA

## Abstract

Pneumatosis intestinalis is a rare but known potential complication of treatment with cetuximab. Here we present two cases of pneumatosis intestinalis occurring in patients who were receiving cetuximab as treatment for advanced head and neck cancer. In both cases, cetuximab was discontinued after discovery of the pneumatosis intestinalis.

## 1. Introduction

Cetuximab is a chimeric monoclonal IgG1 antibody that competitively binds to the epidermal growth factor receptor (EGFR, HER1, and ErbB-1), preventing the binding of the ligand epidermal growth factor and thereby inhibiting phosphorylation and activation of kinase signalling and proliferation pathways. It may also play a role in antibody-dependent cellular cytotoxicity [1]. Cetuximab is used in metastatic colorectal cancer that is KRAS wild type and is being utilized in head and neck cancer in combination with radiation for locally advanced disease, as combination therapy with 5-fluorouracil and platinum-based therapy for recurrent and metastatic disease, or as a single agent in these same patients that have progressed after platinum-based therapy [1, 2]. Common adverse effects include an acneiform rash, hypomagnesemia, and some GI disturbances, although overall it is well tolerated [2].

Pneumatosis intestinalis, the presence of intramural gas in the small or large intestine, is a rare event that has been previously described in patients receiving cetuximab therapy for the treatment of colorectal cancer [3–5], and there has been one other report of this following cetuximab treatment for squamous cell carcinoma of the head and neck [6]. Here we present two cases of pneumatosis intestinalis that occurred while cetuximab was being administered for head and neck cancer.

## 2. Case Reports

### 2.1. Patient 1

**Figure 1:**
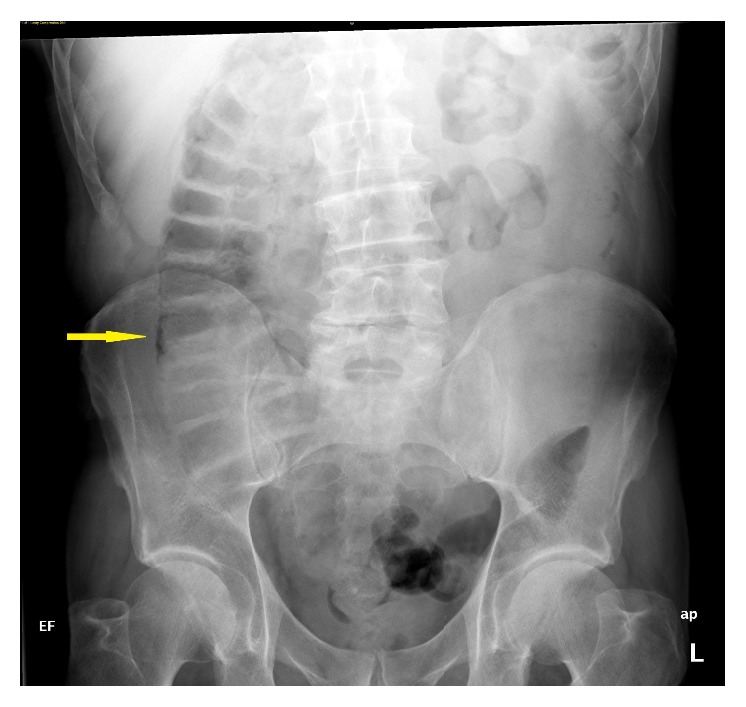
An abdominal X-ray showing pneumatosis intestinalis in the ascending colon of Patient 1.

**Figure 2:**
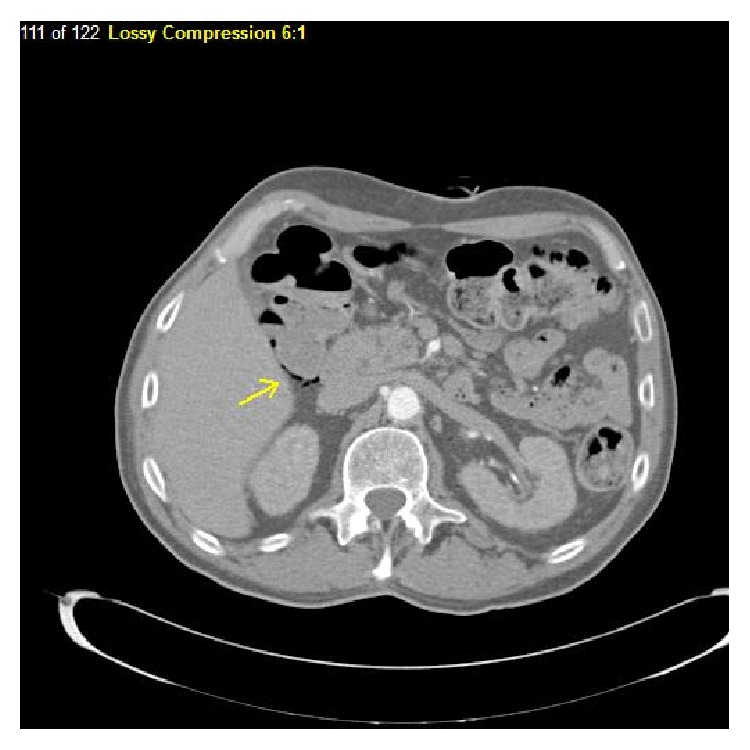
A CT image of dependent air in the bowel wall of Patient 1.

A 71-year-old man was found to have an enlarging neck mass and a subsequent biopsy revealed moderately differentiated squamous cell carcinoma; following induction chemotherapy with docetaxel, carboplatin, and 5-fluorouracil, resection was done. The final pathology of the disease in the neck revealed perineural invasion, lymphovascular space invasion, local extension to the underlying skeletal muscle, and metastasis to 5 of 20 resected lymph nodes. Due to the extensive high risk features, he received radiation with weekly cetuximab. A PEG tube was placed during radiation to the region. Shortly after finishing this course, additional nodes were noted in the neck, and further imaging revealed lung metastasis. The patient was placed on a regimen consisting of paclitaxel, carboplatin, and cetuximab. After disease progression was noted, a salvage regimen of 5-fluorouracil, cisplatin, and cetuximab at a dose of 250 mg/m^2^ was administered. After two cycles of this, his PEG tube needed to be replaced; an abdominal X-ray (Figure 1) following this revealed incidental diffuse pneumatosis intestinalis. A CT scan was obtained to confirm the findings (Figure 2). The patient was asymptomatic at that time, and he was followed conservatively. His chemotherapy and cetuximab were discontinued. Although he did not report any abdominal discomfort or other symptoms that would suggest bowel necrosis, it is unknown if PI resolved as his disease locally in the head and neck and in the lung showed continued progression, and he was eventually referred to Hospice.

### 2.2. Patient 2

**Figure 3:**
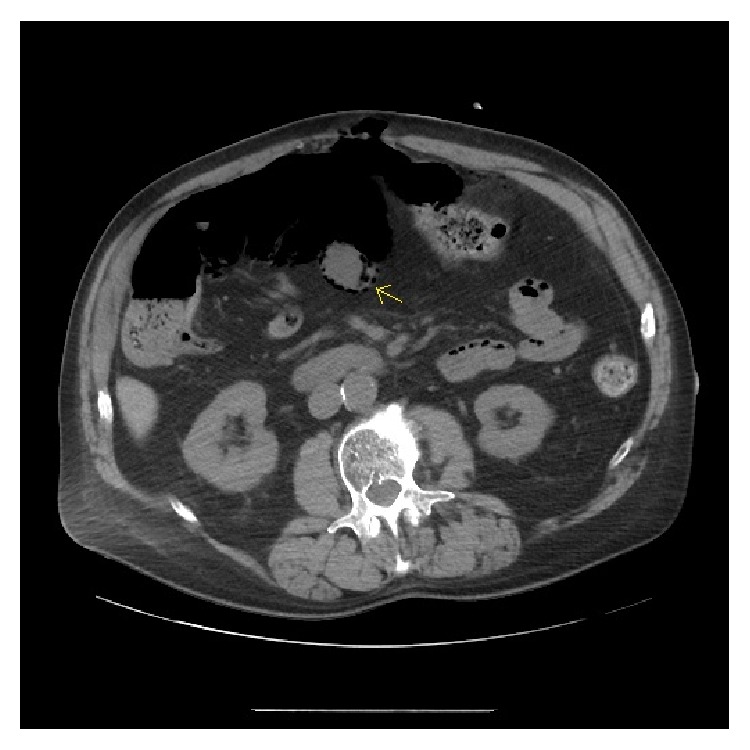
A CT image of dependent air in the bowel wall of Patient 2.

A 66-year-old man with a significant history of cigarette smoking was diagnosed with a squamous cell carcinoma of the left maxillary sinus and he was treated with definitive chemoradiation with cisplatin. Two years following this diagnosis, a lesion of the glottic larynx was noted on endoscopy when the patient had developed a hoarse voice, and the patient underwent definitive chemoradiation to the larynx, again with cisplatin. The following year local disease recurrence was noted and he underwent total laryngectomy and bilateral neck dissection. He then developed local recurrence at the site of the tracheal stoma. He was placed on cetuximab 250 mg/m^2^ and appeared to have a good local response. Resection of the tracheal stomal recurrence was then attempted, and, after a multidisciplinary meeting, it was decided that he may benefit from a modified radiation treatment locally to the region of the stoma with weekly cetuximab. During this course, the patient developed upper abdominal discomfort, and imaging revealed pneumatosis intestinalis (Figure 3). He was admitted to the hospital due to his symptomatology, and general surgery was consulted. He was placed on bowel rest and intravenous antibiotics. After approximately 2 weeks, his abdominal discomfort improved and follow-up imaging revealed resolving pneumatosis intestinalis. Enteral feeding was restarted, and antibiotics were discontinued. Follow-up CT imaging two months later confirmed resolution of pneumatosis intestinalis.

## 3. Discussion

Pneumatosis intestinalis refers to the presence of gas in cysts within the submucosa or subserosa of the gastrointestinal tract. It is not a disease per se, but it is considered to be a sign of some underlying disorder, although it continues to be idiopathic in approximately 15% of cases [7]. Possible causes include infiltration of gas alongside mesenteric vessels perhaps originating from the lung via the mediastinum, dissection of gas into the bowel wall from the intestinal lumen, and production of gas either after infiltration of bacteria through the mucosa or by increased gas pressure within the lumen due to bacterial overgrowth or medications that cause excess intestinal gas [7, 8]. It has a variable course, ranging from recovery with conservative treatment including treatment of the underlying etiology to death. The presence of pneumatosis intestinalis has been linked to mucosal disruption, infections, COPD, gastrointestinal motility disorders, iatrogenic causes such as endoscopic procedures, and immune compromise, including administration of chemotherapy. Previously, cyclophosphamide, cytarabine, docetaxel, irinotecan, cisplatin, 5-fluorouracil, and bleomycin have been linked to the development of pneumatosis intestinalis [7]. More recently, PI has also been seen in molecularly targeted agents, including bevacizumab and sunitinib [9].

Patient 1 had an upper endoscopic procedure for a feeding tube placement 6 months prior to the discovery of pneumatosis intestinalis, and this is unlikely to have been a causative etiology. He was receiving 5-fluorouracil as part of his cetuximab containing regimen at the time of his diagnosis. Patient 2 also had undergone an upper endoscopic procedure for feeding tube placement, 5 months before his complaints, and had no other attributable risk factors for pneumatosis intestinalis.

## 4. Conclusion

The usage of biologically targeted agents in the treatment of cancer is likely to increase, especially in the molecular era of cancer and personalized medicine. As these agents are increasingly used, the potential to give active agents that seem to have a more tolerable side effect profile as compared with chemotherapy while still providing meaningful clinical impact is attractive. These cases illustrate the need to be aware of adverse effects that differ from those traditionally seen with cytotoxic chemotherapy.
